# Association of Different Definitions of Erythropoiesis-Stimulating Agent Hyporesponsiveness with Major Adverse Cardiovascular Events: Insights from ASCEND-D

**DOI:** 10.34067/KID.0000000808

**Published:** 2025-05-07

**Authors:** Finnian R. McCausland, Sushrut S. Waikar, Brian Claggett, Gearoid M. McMahon, Osvaldo M.V. Neto, Anjay Rastogi, Kearkiat Praditpornsilpa, Ricardo Correa-Rotter, Vijay Kher, Lucia Del Vecchio, Scott D. Solomon, Ajay K. Singh

**Affiliations:** 1 Brigham and Women's Hospital, Harvard Medical School, Boston, Massachusetts; 2 Boston Medical Center, Boston University School of Medicine, Boston, Massachusetts; 3 Serviço de Nefrologia de Ribeirao Preto, São Paulo, Brazil; 4 David Geffen School of Medicine at UCLA, Los Angeles, California; 5 King Chulalongkorn Memorial Hospital, Chulalongkorn University, Bangkok, Thailand; 6 Instituto Nacional de Ciencias Médicas y Nutrición Salvador Zubirán, Mexico City, Mexico; 7 Epitome Kidney & Urology Institute, New Delhi, India; 8 Sant'Anna Hospital, ASST Lariana, Como, Italy

**Keywords:** anemia, cardiovascular events, chronic dialysis, chronic hemodialysis, clinical trial, erythropoietin, hypoxia

## Abstract

**Key Points:**

- Among patients receiving maintenance dialysis, an inadequate hemoglobin response to erythropoiesis-stimulating agents (ESAs) is associated with a higher risk of adverse outcomes.
- Among patients in ASCEND-D, all three different prespecified definitions of ESA hyporesponsiveness were similarly associated with major adverse cardiovascular event outcomes.
- ESA hyporesponsiveness should be considered an important clinical parameter for risk-stratifying patients with kidney failure requiring dialysis.

**Background:**

Hyporesponsiveness to erythropoiesis-stimulating agents (ESAs) is a common clinical problem and is associated with major adverse cardiovascular events (MACEs). Although several definitions have been proposed, data examining associations with MACE in clinical trials are limited.

**Methods:**

Anemia Studies in Chronic Kidney Disease: Erythropoiesis via a Novel Prolyl Hydroxylase Inhibitor Daprodustat-Dialysis (ASCEND-D, NCT02879305), a large event-driven cardiovascular outcomes trial, randomized 2964 patients receiving maintenance dialysis to either daprodustat or conventional ESAs. All patients received an ESA for at least 6 weeks before randomization and were managed with dosing algorithms for iron and randomized treatment. Three definitions of ESA hyporesponsiveness were prespecified: (*1*) ESA hyporesponsiveness definition 1 (HypoR1): an erythropoietin resistance index ≥2 U/kg per week per gram per liter or prior ESA dose/estimated dry weight ≥450 U/kg per week, (*2*) ESA hyporesponsiveness definition 2 (HypoR2): erythropoietin resistance index ≥1.5 U/kg per week per gram per liter, and (*3*) ESA hyporesponsiveness definition 3 (HypoR3): baseline ESA dose (U/wk) in top 20th percentile. Adjusted Cox regression models were fit to examine the association of each definition with the adjudicated MACE composite (death, nonfatal myocardial infarction, and nonfatal stroke).

**Results:**

Baseline ESA hyporesponsiveness was present in 12%, 20%, and 20% of patients according to definitions HypoR1, HypoR2, and HypoR3, respectively. Compared with those without hyporesponsiveness, all definitions were associated with a higher risk of the composite MACE outcome: adjusted hazard ratio (HR) 1.32 (95% confidence interval [CI], 1.04 to 1.68) for HypoR1, HR 1.33 (95% CI, 1.08 to 1.63) for HypoR2, and HR 1.36 (95% CI, 1.12 to 1.66) for HypoR3. There was no evidence for effect modification by randomized treatment (*P* interaction > 0.40 for all).

**Conclusions:**

Baseline ESA hyporesponsiveness is a potent predictor of MACE among patients receiving maintenance dialysis in ASCEND-D. All prespecified definitions were similarly associated with a higher risk of MACE.

**Clinical Trial registry name and registration number:**

NCT02879305.

## Visual Abstract

**Figure:**
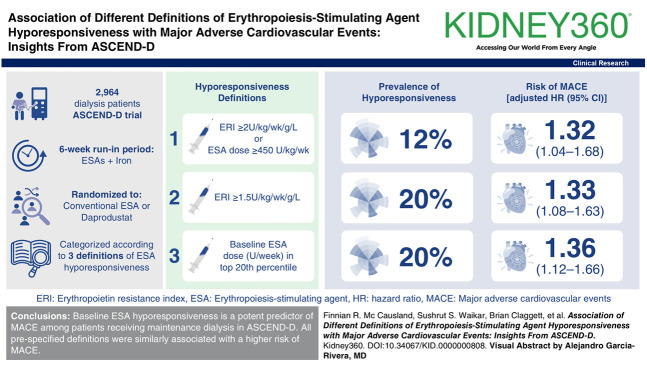

## Introduction

The development of recombinant human erythropoietin revolutionized the treatment of anemia of CKD, providing an outpatient parenteral therapy that could increase hemoglobin and reduce the need for blood transfusions.^1–3^

Early reports observed a dose-response relationship for erythropoiesis-stimulating agents (ESAs), with higher ESA doses generally resulting in a faster rate of increase or higher overall hemoglobin concentration.^1,3^ Conversely, subgroups of patients were identified who failed to achieve target hemoglobin concentrations in response to typical ESA dosing. Although the etiology of this phenomenon remains incompletely understood,^4,5^ ESA hyporesponsiveness seems to have prognostic implications, carrying a higher associated risk for patient morbidity and mortality, compared with “normal” responsiveness comparators.^6–13^

ESA hyporesponsiveness is estimated to affect approximately 13%–30% of patients receiving maintenance hemodialysis, although it varies somewhat according to the definition used.^8,9,14^ While most consider hemoglobin changes to weight-based doses of ESA, there is little consensus on a preferred definition in contemporary practice,^4^ which hinders clinical applicability and comparisons between studies. In this study, we analyzed the association of three prespecified definitions of ESA hyporesponsiveness with major adverse cardiovascular event (MACE) among patients in the Anemia Studies in Chronic Kidney Disease: Erythropoiesis via a Novel Prolyl Hydroxylase Inhibitor Daprodustat-Dialysis (ASCEND-D) trial.

## Methods

### Study Patients

The design, methods, and primary results of ASCEND-D have been published.^15,16^ In brief, ASCEND-D enrolled adult patients who had received maintenance dialysis for at least 90 days, had been treated with an ESA for at least 6 weeks, were adherent to daprodustat placebo tablets during run-in, and who met the trial hemoglobin requirements (8–11 g/dl). Major exclusion criteria included ferritin ≤100 *μ*g/L and transferrin saturations ≤20% at screening; evidence of nonrenal anemia; uncontrolled hypertension; myocardial infarction (MI), acute coronary syndrome, stroke, or transient ischemic attack within 4 weeks of screening; and New York Heart Association Class 4 heart failure. Local ethics committees approved the trial, and all patients provided written informed consent. The study was conducted in accordance with the principles of the Declaration of Helsinki.

### Trial Procedures

Eligible patients were randomized in a 1:1 ratio and open-label manner to either oral daprodustat or an injectable ESA (intravenous epoetin alfa for those receiving hemodialysis and subcutaneous darbepoetin alfa for those receiving peritoneal dialysis), stratified by dialysis type, geographic region, and participation in a BP monitoring substudy. Trial-specific dosing algorithms were used to achieve and maintain a target hemoglobin of 10–11 g/dl. An iron management algorithm and rescue algorithm (including provision for intravenous iron or red cell transfusion) were also implemented.^16^

### Exposures of Interest: Prespecified Definitions of Baseline ESA Hyporesponsiveness

For each subject, the baseline erythropoietin resistance index (ERI) was calculated as the dry weight adjusted weekly mean ESA dose during the 8 week screening period divided by baseline hemoglobin concentration. Three definitions of baseline ESA hyporesponsiveness were prespecified in the statistical analysis plan and were considered as the exposures of interest for the present analyses^16^: definition 1 (HypoR1): ERI ≥2 U/kg per week per gram per liter or prior ESA dose/estimated dry weight ≥450 U/kg per week, definition 2 (HypoR2): ERI ≥1.5 U/kg per week per gram per liter, and definition 3 (HypoR3): baseline ESA dose (U/wk) in top 20th percentile. Data on baseline ERI were not available for 38 of the original 2964 (1.3%) participants, who were excluded from further analyses.

### Outcomes of Interest

The primary outcome for the present analyses was the first occurrence of a composite MACE (death from any cause, nonfatal MI, and nonfatal stroke). An independent end point committee adjudicated all cardiovascular events in a blinded fashion.

### Statistical Analysis

Data were reported as mean (±SD) when normally distributed, as median (25th–75th percentile) when non-normally distributed, and as frequencies and percentages for categorical variables. Differences in baseline characteristics were assessed by the Student *t* test, Wilcoxon rank-sum test, or chi-squared test for trend for continuous normal, continuous non-normal, and categorical data, respectively.

Incidence rates and 95% confidence intervals (CIs) were calculated per 100 patient-years of follow-up. Cox proportional hazards models were fit to estimate the association of ESA hyporesponsiveness with the primary composite MACE outcome. These models were adjusted for randomized treatment assignment (daprodustat versus conventional ESA), geographic region (Asia Pacific; Eastern Europe/South Africa; Western Europe, Canada, Australia, and New Zealand; Latin America; and the United States), age (linear), sex (self-reported male or female), race (self-reported American Indian or Alaska Native, Asian, Black or African American, multiple, native Hawaiian or other Pacific Islander, and White), diabetes, history of cardiovascular disease (CV), smoking status (current, former, never), categories of dialysis vintage (0 to <2, 2 to <5, ≥5 years), dialysis access (arteriovenous fistula, arteriovenous graft, venous catheter, and peritoneal catheter), body mass index (linear), systolic BP (linear), baseline concentrations of hemoglobin (linear), serum albumin (linear), log-transformed serum intact parathyroid (linear), log-transformed high-sensitivity C-reactive protein (linear), and Kt/V (linear). The variables selected for inclusion in the multivariable model were chosen based on clinical and biologic plausibility, as well as a review of prior literature. Interaction terms were included to assess if the association of ESA hyporesponsiveness with the composite MACE outcome differed according to the randomized treatment assignment. In an exploratory analysis, the relative strength of the associations of individual variables (including hyporesponder definition HypoR1) with the MACE outcome were assessed through Z-scores. This was also performed with a backward selection model using a *P* value for retention in the model of <0.05.

In further exploratory analyses, restricted cubic splines were fit to assess the adjusted association of log-transformed baseline ERI with MACE. The reference was adjusted to explore a threshold ERI value beyond which higher values did not have significantly higher associations with MACE outcomes.

All analyses were performed at an *α* level of 0.05, without correction for multiple hypothesis testing using Stata MP (version 18.0, Stata Corp., College Station, TX).

## Results

### Patient Characteristics

Of the 2926 patients included in the analyses, the mean age was 57±14 years, 43% were female, and 89% received hemodialysis as their maintenance therapy. The proportion of patients meeting the criteria for ESA hyporesponsiveness was 12% for definition HypoR1, 20% for definition HypoR2, and 20% for definition HypoR3. Of the 581 patients meeting the HypoR3 definition, 464 (80%) also met the HypoR2 definition, while 343 (59%) also met the HypoR1 definition (Supplemental Figure 1).

Patients meeting definition HypoR1 criteria for ESA hyporesponsiveness tended to be younger, female, non-White, of longer dialysis vintage, receive peritoneal dialysis, and have lower post-dialysis body mass index, serum albumin, transferrin saturation, and hemoglobin concentrations. Patients with ESA hyporesponsiveness were more likely than those without to have higher systolic BP, high-sensitivity C-reactive protein concentrations, and a higher standardized prior ESA dose (Table 1). Differences in baseline characteristics according to ESA hyporesponsiveness definitions HypoR2 and HypoR3 are presented in Supplemental Tables 1 and 2, respectively.

**Table 1:** Baseline characteristics according to erythropoiesis-stimulating agent hyporesponsiveness status (definition HypoR1)^a^

Characteristic	Non-ESA Hyporesponsiveness (*n*=2563)	ESA Hyporesponsiveness (*n*=363)	*P*-value
Age, yr	58±14	53±15	*P* < 0.001
Female, *n* (%)	1074 (41.9)	184 (50.7)	*P* = 0.002
**Race, *n* (%)**			*P* < 0.001
American Indian or Alaska Native	28 (1.1)	15 (4.1)	
Asian	289 (11.3)	62 (17.1)	
Black or African American	379 (14.8)	68 (18.7)	
Multiple	49 (1.9)	13 (3.6)	
Native Hawaiian or other Pacific Islander	43 (1.7)	8 (2.2)	
White	1775 (69.3)	197 (54.3)	
**Dialysis vintage, yr, *n* (%)**			*P* = 0.05
0 to <2	794 (31.0)	91 (25.1)	
2 to <5	917 (35.8)	134 (36.9)	
≥5	852 (33.2)	138 (38.0)	
Hemodialysis, *n* (%)	2292 (89.4)	308 (84.8)	*P* = 0.01
**Access type, *n* (%)**			*P* = 0.003
AVF	1827 (71.4)	227 (62.5)	
AVG	221 (8.6)	34 (9.4)	
Central venous catheter	242 (9.5)	47 (12.9)	
Peritoneal catheter	270 (10.5)	55 (15.2)	
Postdialysis BMI at baseline, kg/m^2^	27 (23–32)	25 (21–29)	*P* < 0.001
Systolic BP, mm Hg	133±21	139±24	*P* < 0.001
History of diabetes, *n* (%)	1071 (41.8)	142 (39.1)	*P* = 0.33
History of CV, *n* (%)	1175 (45.8)	152 (41.9)	*P* = 0.15
**Smoking status, *n* (%)**			*P* = 0.17
Current	235 (9.2)	23 (6.3)	
Former	541 (21.1)	84 (23.1)	
Never	1787 (69.7)	256 (70.5)	
Hemoglobin, g/dl	10.4±0.9	9.9±1.0	*P* < 0.001
Albumin, g/dl	3.9±0.4	3.7±0.4	*P* < 0.001
iPTH, ng/L	316 (158–567)	347 (144–649)	*P* = 0.13
hsCRP, mg/L	3.7 (1.5–9.8)	6.0 (2.2–14.9)	*P* < 0.001
Standardized prior ESA dose, U/wk	5207 (3016–8000)	17,787 (15,714–24,137)	*P* < 0.001
Ferritin, ng/ml	597 (347–954)	591 (313–969)	*P* = 0.61
Transferrin saturation, %	33 (26–42)	30 (23–38)	*P* < 0.001
Kt/V	1.59±0.38	1.64±0.44	*P* = 0.03
Randomized to daprodustat, *n* (%)	1284 (50.1)	183 (50.4)	*P* = 0.91

AVF, ateriovenous fistula; AVG, arteriovenous graft; BMI, body mass index; CV, cardiovascular disease; ESA, erythropoiesis-stimulating agent; hsCRP, high sensitivity C-reactive protein; HypoR1, ESA hyporesponsiveness definition 1; iPTH, intact parathyroid hormone.

a Definition HypoR1—an erythropoietin resistance index (calculated as the weight-adjusted weekly erythropoiesis-stimulating agent dose, divided by the hemoglobin) of ≥2 U/kg per week per gram per liter or prior erythropoiesis-stimulating agent dose/estimated dry weight ≥450 U/kg per week.

### ESA Hyporesponsiveness and the MACE Composite Outcome

**Figure 1:**
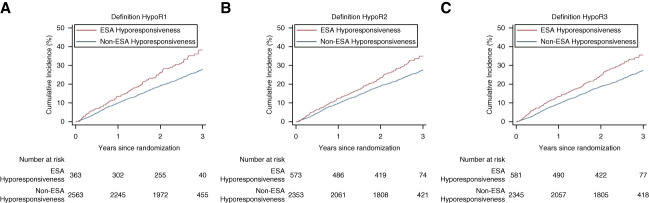
**Kaplan-Meier plots of time to first occurrence of adjudicated MACE according to ESA hyporesponsiveness status definitions.** Shown is the cumulative incidence of a first adjudicated MACE (a composite of death from any cause, nonfatal MI, or nonfatal stroke) according to ESA hyporesponsiveness definitions. Definition HypoR1: an ERI ≥2 U/kg per week per gram per liter or prior ESA dose/estimated dry weight ≥450 U/kg per week (A). Definition HypoR2: ERI ≥1.5 U/kg per week per gram per liter (B). Definition HypoR3: baseline ESA dose (U/wk) in top 20th percentile (C). ERI, erythropoietin resistance index; ESA, erythropoiesis-stimulating agent; HypoR1, ESA hyporesponsiveness definition 1; HypoR2, ESA hyporesponsiveness definition 2; HypoR3, ESA hyporesponsiveness definition 3; MACE, major adverse cardiovascular event; MI, myocardial infarction.

In unadjusted analyses, all definitions of ESA hyporesponsiveness were associated with a higher risk of developing the MACE outcome, compared with non-ESA hyporesponsiveness (Figure 1 and Supplemental Table 3).

Effect estimates were attenuated following multivariable adjustment for all definitions of ESA hyporesponsiveness considered, with a 32% higher risk with definition HypoR1 of ESA hyporesponsiveness (hazard ratio [HR], 1.32; 95% CI, 1.04 to 1.68), 33% higher risk with definition HypoR2 of ESA hyporesponsiveness (HR, 1.33; 95% CI, 1.08 to 1.63), and 36% higher risk with definition HypoR3 of ESA hyporesponsiveness (HR, 1.36; 95% CI, 1.12 to 1.66; Table 2). The results of the full multivariable model for definition HypoR1 are presented in Supplemental Table 4.

**Table 2:** Major adverse cardiovascular events according to erythropoiesis-stimulating agent hyporesponsiveness status^a^

Outcome	ESA Hyporesponsiveness		Non-ESA Hyporesponsiveness		Adjusted HR (95% CI)^b^	*P* Value
	No. Events/No. Patients (%)	Rate/100 Patient-Years (95% CI)	No. Events/No. Patients (%)	Rate/100 Patient-Years (95% CI)		
**Definition HypoR1**						
MACE composite	117/363 (32)	15.3 (12.8 to 18.3)	635/2563 (25)	10.8 (10.0 to 11.7)	1.32 (1.04 to 1.68)	0.02
*All-cause death*	96/363 (26)	11.9 (9.7 to 14.5)	485/2563 (19)	7.9 (7.2 to 8.6)	1.39 (1.06 to 1.82)	0.02
*Nonfatal MI*	35/363 (10)	4.5 (3.3 to 6.3)	197/2563 (8)	3.3 (2.9 to 3.8)	1.39 (0.92 to 2.09)	0.11
*Nonfatal stroke*	7/363 (2)	0.9 (0.4 to 1.8)	62/2563 (2)	1.0 (0.8 to 1.3)	1.13 (0.49 to 2.61)	0.77
**Definition HypoR2**						
MACE composite	174/573 (30)	14.0 (12.0 to 16.2)	578/2353 (25)	10.7 (9.9 to 11.6)	1.33 (1.08 to 1.63)	0.01
*All-cause death*	142/573 (25)	10.9 (9.2 to 12.8)	439/2353 (19)	7.8 (7.1 to 8.5)	1.37 (1.09 to 1.73)	0.01
*Nonfatal MI*	52/573 (9)	4.2 (3.2 to 5.4)	180/2353 (8)	3.3 (2.8 to 3.8)	1.55 (1.10 to 2.20)	0.01
*Nonfatal stroke*	9/573 (2)	0.7 (0.4 to 1.3)	60/2353 (3)	1.1 (0.8 to 1.4)	0.91 (0.43 to 1.92)	0.80
**Definition HypoR3**						
MACE composite	183/581 (31)	14.6 (12.6 to 16.8)	569/2345 (24)	10.5 (9.7 to 11.5)	1.36 (1.12 to 1.66)	0.002
*All-cause death*	145/581 (25)	10.9 (9.2 to 12.8)	436/2345 (19)	7.7 (7.0 to 8.5)	1.41 (1.12 to 1.76)	0.003
*Nonfatal MI*	56/581 (10)	4.4 (3.4 to 5.7)	176/2345 (8)	3.2 (2.8 to 3.7)	1.42 (1.02 to 1.99)	0.04
*Nonfatal stroke*	12/581 (2)	0.9 (0.5 to 1.6)	57/2345 (2)	1.0 (0.8 to 1.3)	1.15 (0.59 to 2.25)	0.68

CI, confidence interval; ESA, erythropoiesis-stimulating agent; HR, hazard ratio; HypoR1, ESA hyporesponsiveness definition 1; HypoR2, ESA hyporesponsiveness definition 2; HypoR3, ESA hyporesponsiveness definition 3; MACE, major adverse cardiovascular event; MI, myocardial infarction.

a Definition HypoR1—an erythropoietin resistance index (calculated as the weight-adjusted weekly erythropoiesis-stimulating agent dose, divided by the hemoglobin) of ≥2 U/kg per week per gram per liter or prior erythropoiesis-stimulating agent dose/estimated dry weight ≥450 U/kg per week; definition HypoR2—erythropoietin resistance index ≥1.5 U/kg per week per gram per liter; definition HypoR3—baseline erythropoiesis-stimulating agent dose (U/wk) in top 20th percentile.

b Models were adjusted for randomized treatment assignment, baseline dialysis modality (hemodialysis or peritoneal dialysis), geographic region, age, sex, race, diabetes, history of cardiovascular disease, smoking status (current, former, never), categories of dialysis vintage (0 to <2, 2 to <5, ≥5 years), body mass index, systolic BP, baseline concentrations of hemoglobin, serum albumin, log-transformed serum intact parathyroid, and log-transformed high sensitivity C-reactive protein.

In exploratory analyses using restricted cubic splines, the association of log-transformed baseline ERI with MACE outcomes seemed to be linear. A reference value equivalent to a baseline ERI value of 1.28 U/kg per week per gram per deciliter seemed to be a point of inflection (*i.e*., compared with this reference, higher values were not significantly associated with a higher risk of MACE, but values below this were associated with a lower risk (Supplemental Figure 2).

### ESA Hyporesponsiveness and Individual Components of the MACE Outcome

In unadjusted analyses, all three definitions of ESA hyporesponsiveness were associated with a higher risk of all-cause mortality. The association with nonfatal MI varied by definition, while none were significantly associated with a higher risk of nonfatal stroke (Supplemental Table 3).

In adjusted analyses, all three definitions of ESA hyporesponsiveness were attenuated but remained associated with a higher risk of all-cause mortality. Although the most potent effect estimate was observed with definition HypoR3 (HR, 1.41; 95% CI, 1.12 to 1.76), all three risk estimates were quantitatively similar (Table 2). Regarding the outcome of nonfatal MI, the most potent effect estimate was observed for definition HypoR2 (HR, 1.55; 95% CI, 1.10 to 2.20). Although the numerical spread of effect estimates for the three definitions was wider, the CIs were relatively large (Table 2). There was no association of any definition of ESA hyporesponsiveness with nonfatal stroke (Supplemental Table 3).

### ESA Hyporesponsiveness and MACE Outcomes According to Randomized Treatment Arm

**Figure 2:**
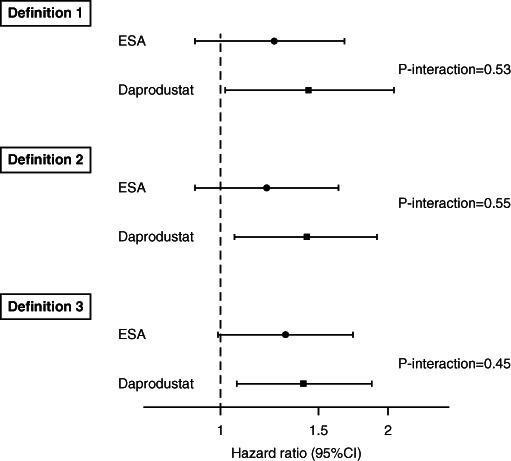
**Adjusted association of ESA hyporesponsiveness with MACE according to randomized treatment arm.** CI, confidence interval.

Interaction terms were added to the multivariable model to assess for the potential differential associations of baseline ESA hyporesponsiveness with the MACE composite outcome, according to the randomized treatment arm. The *P* values for interaction were 0.67, 0.41, and 0.60 for definitions 1, 2, and 3 of ESA hyporesponsiveness, respectively (Figure 2).

### Exploration of the Strength of the Association of Multivariable Model Components with the MACE Outcome

The strength of the association of individual variables of the multivariable model with the MACE outcome was assessed through Z-scores in both the fully adjusted model and a stepwise model (Supplemental Table 4). In both approaches, the most potent predictors were age, history of CV, and serum albumin.

## Discussion

Among patients receiving maintenance dialysis enrolled in the ASCEND-D trial, all three prespecified definitions of baseline ESA hyporesponsiveness were independently associated with a higher risk of subsequent MACE outcomes, with broadly similar adjusted point estimates. There was no suggestion of differential associations according to the randomized treatment arm.

ESA hyporesponsiveness can be broadly defined as a failure to increase hemoglobin in response to typical ESA doses or the requirement of higher ESA doses to achieve a particular hemoglobin concentration.^4^ Further granularity was incorporated by Kidney Disease Improving Global Outcomes and National Institute for Health and Care Excellence guidelines,^17,18^ which considered ESA doses above that of the maximum recommended weight-based starting dose, while the ERI (used in definitions HypoR1 and HypoR2 of the present analyses) examines thresholds of a weight-adjusted weekly ESA dose, indexed to the hemoglobin concentration. As practice patterns for the treatment of anemia among patients receiving maintenance hemodialysis have changed over the last two decades, including lower overall ESA doses and greater use of intravenous iron, the nuances of the different definitions are highly relevant. However, to date, there is no consensus on the optimal definition of ESA hyporesponsiveness among patients receiving maintenance dialysis.

The prevalence of ESA hyporesponsiveness is dependent on the definition used and the patient population considered, ranging from 12.5% among patients receiving maintenance hemodialysis (when defined as two consecutive hemoglobin measurements <10 g/dl with ESA dose >7700 U/treatment)^8^ to 30% among patients with CKD or cancer (when defined as any change in hemoglobin <0 g/dl over 6 months from initiation of ESA).^14^ In our analyses, differences in baseline prevalence were also observed, ranging from 12% with definition HypoR1 to 20% for definition HypoR2, with the latter reflecting a less stringent criterion for ESA hyporesponsiveness. Of note, the prevalence using definition HypoR1 in our analyses is much lower than the 53% reported from United States Renal Data System data using the same ERI definition.^9^ It is important to note differences in the patient population, with the United States Renal Data System study having an older population with a markedly higher comorbid disease burden, which may partially explain the observed differences.

Our data are broadly consistent with previous studies that reported higher risks of adverse outcomes with various definitions of ESA hyporesponsiveness.^6–14^ We observed that all three prespecified definitions of ESA hyporesponsiveness had similar magnitudes of association with the composite MACE outcome (noting that there was 80% overlap of patients between hyporesponder definitions HypoR2 and HypoR3). This is consistent with other reports that observed higher risks of adverse outcomes with the highest, compared with the lowest tertile, of the ERI metric.^13^ Indeed, in spline analyses, we observed a linear association of higher baseline ERI with MACE outcomes, suggesting that there is likely a continuum of associated risk. Although our data confirm that ESA hyporesponsiveness is an important prognostic marker, whether the association of ESA hyporesponsiveness with a higher risk of MACE reflects a sicker patient population (*e.g*., more comorbidities, inflammation)^19,20^ or is a marker of adverse sequelae of higher doses of ESA^21^ remains unclear.

The effect estimates for the all-cause death and nonfatal MI components of the MACE composite were directionally consistent with the main composite, whereas we did not observe a higher risk for nonfatal stroke with any of the prespecified definitions of ESA hyporesponsiveness. This is in contrast to some prior reports that did observe a higher risk of stroke.^9^ Potential explanations for the lack of association with stroke in our analyses may include differences in contemporary dosing, patient characteristics, or simply a lack of power from the relative paucity of stroke events in the ASCEND-D follow-up period.

The etiology of ESA hyporesponsiveness is likely multifactorial, with contributions from inflammation, iron deficiency, hyperparathyroidism, and inadequate clearance from dialysis, among others.^4,5^ In the present analyses, the most potent associations for MACE outcomes in multivariable models were observed with age, history of CV disease, and lower serum albumin. However, even after adjusting for these and other factors, we still observed an association of baseline ESA hyporesponsiveness with adverse outcomes. With potentially beneficial attributes related to iron metabolism and hepcidin concentration^22^ and lower peak serum erythropoietin concentration,^23^ it was hoped that hypoxia-inducible factor-prolyl hydroxylase inhibitors (HIF-PHIs) might provide viable alternative therapies for the treatment of anemia of CKD, particularly among ESA hyporesponsive patients.^24^ In our analyses, we did not find evidence for differential associations of baseline ESA hyporesponsiveness with MACE outcomes according to randomized treatment arm. However, the question of whether HIF-PHIs are advantageous (versus conventional ESAs) in ESA hyporesponsive patients, either for increasing hemoglobin or have lower risk of MACE, remains unclear. To address this specific question, adequately powered trials examining the effect of HIF-PHIs versus conventional ESAs among the specific subgroup of ESA hyporesponsive patients are needed.

The strengths of our study include the use of prespecified definitions of ESA hyporesponsiveness and the availability of adjudicated MACE outcomes in the setting of a randomized controlled trial. However, there are some limitations to our analyses, which include the relative paucity of stroke events and the potential for residual confounding, despite the use of multivariable-adjusted modeling approaches. ESA hyporesponsiveness was only defined at baseline and daprodustat dosing has not been validated for hyporesponsiveness equivalency, precluding the examination of time-updated associations of ESA hyporesponsiveness with outcomes. A dedicated trial recruiting ESA hyporesponsive patients would be needed to examine if daprodustat or conventional ESAs result in better clinical outcomes in this specific population. Further limitations relate to lack of objective data on cardiac structure and function and the generalizability of the results beyond that of the included patient population.

In conclusion, all three prespecified definitions of baseline ESA hyporesponsiveness in the ASCEND-D trial were similarly associated with a higher risk of MACE outcomes and did not differ according to the randomized treatment arm.

## Supplementary Material

## Data Availability

Partial restrictions to the data and/or materials apply. Anonymized individual patient data and study documents can be requested for further research from www.clinicalstudydatarequest.com.

## Supplemental Material

Supplemental Table 1. Baseline characteristics according to ESA hyporesponsiveness status (definition HypoR2).

Supplemental Table 2. Baseline characteristics according to ESA hyporesponsiveness status (definition HypoR3).

Supplemental Table 3. MACE according to ESA hyporesponsiveness status (Unadjusted analyses).

Supplemental Table 4. Multivariable adjusted association of ESA hyporesponsiveness (definition 1) with MACE.

Supplemental Figure 1. Venn diagram illustrating overlap of patients between three definitions of ESA hyporesponsiveness.

Supplemental Figure 2. Restricted cubic spline showing the adjusted association of baseline log-transformed ERI with risk of MACE outcomes.

## References

[1] Eschbach JW Egrie JC Downing MR Browne JK Adamson JW. Correction of the anemia of end-stage renal disease with recombinant human erythropoietin. Results of a combined phase I and II clinical trial. New Engl J Med. 1987;316(2):73–78. doi:10.1056/NEJM198701083160203 3537801

[2] Eschbach JW Abdulhadi MH Browne JK, . Recombinant human erythropoietin in anemic patients with end-stage renal disease. Results of a phase III multicenter clinical trial. Ann Intern Med. 1989;111(12):992–1000. doi:10.7326/0003-4819-111-12-992 2688507

[3] Winearls C Oliver DO Pippard MJ Reid C Downing MR Cotes PM. Effect of human erythropoietin derived from recombinant DNA on the anaemia of patients maintained by chronic haemodialysis. Lancet. 1986;2(8517):1175–1178. doi:10.1016/s0140-6736(86)92192-6 2877323

[4] Weir MR. Managing anemia across the stages of kidney disease in those hyporesponsive to erythropoiesis-stimulating agents. Am J Nephrol. 2021;52(6):450–466. doi:10.1159/000516901 34280923

[5] Macdougall IC Meadowcroft AM Blackorby A, . Regional variation of erythropoiesis-stimulating agent hyporesponsiveness in the global daprodustat dialysis study (ASCEND-D). Am J Nephrol. 2023;54(1-2):1–13. doi:10.1159/000528696 36739866 PMC10210075

[6] Kainz A Mayer B Kramar R Oberbauer R. Association of ESA hypo-responsiveness and haemoglobin variability with mortality in haemodialysis patients. Nephrol Dial Transplant. 2010;25(11):3701–3706. doi:10.1093/ndt/gfq287 20507852 PMC3360143

[7] Solomon SD Uno H Lewis EF, .; Trial to Reduce Cardiovascular Events with Aranesp Therapy (TREAT) Investigators. Erythropoietic response and outcomes in kidney disease and type 2 diabetes. New Engl J Med. 2010;363(12):1146–1155. doi:10.1056/NEJMoa1005109 20843249

[8] Luo J Jensen DE Maroni BJ Brunelli SM. Spectrum and burden of erythropoiesis-stimulating agent hyporesponsiveness among contemporary hemodialysis patients. Am J Kidney Dis. 2016;68(5):763–771. doi:10.1053/j.ajkd.2016.05.031 27528373

[9] Cizman B Smith HT Camejo RR, . Clinical and economic outcomes of erythropoiesis-stimulating agent hyporesponsiveness in the post-bundling era. Kidney Med. 2020;2(5):589–599.e1. doi:10.1016/j.xkme.2020.06.008 33089137 PMC7568064

[10] Kilpatrick RD Critchlow CW Fishbane S, . Greater epoetin alfa responsiveness is associated with improved survival in hemodialysis patients. Clin J Am Soc Nephrol. 2008;3(4):1077–1083. doi:10.2215/CJN.04601007 18417744 PMC2440273

[11] Bradbury BD Danese MD Gleeson M Critchlow CW. Effect of epoetin alfa dose changes on hemoglobin and mortality in hemodialysis patients with hemoglobin levels persistently below 11 g/dL. Clin J Am Soc Nephrol. 2009;4(3):630–637. doi:10.2215/CJN.03580708 19261826 PMC2653654

[12] Zhang Y Thamer M Stefanik K Kaufman J Cotter DJ. Epoetin requirements predict mortality in hemodialysis patients. Am J Kidney Dis. 2004;44(5):866–876. doi:10.1016/s0272-6386(04)01086-8 15492953

[13] Okazaki M Komatsu M Kawaguchi H Tsuchiya K Nitta K. Erythropoietin resistance index and the all-cause mortality of chronic hemodialysis patients. Blood Purif. 2014;37(2):106–112. doi:10.1159/000358215 24603656

[14] Ingrasciotta Y Lacava V Marcianò I, . In search of potential predictors of erythropoiesis-stimulating agents (ESAs) hyporesponsiveness: a population-based study. BMC Nephrol. 2019;20(1):359. doi:10.1186/s12882-019-1554-0 31521117 PMC6744676

[15] Singh AK Blackorby A Cizman B, . Study design and baseline characteristics of patients on dialysis in the ASCEND-D trial. Nephrol Dial Transplant. 2022;37(5):960–972. doi:10.1093/ndt/gfab065 33744933 PMC9035347

[16] Singh AK Carroll K Perkovic V, .; ASCEND-D Study Group. Daprodustat for the treatment of anemia in patients undergoing dialysis. New Engl J Med. 2021;385(25):2325–2335. doi:10.1056/NEJMoa2113379 34739194

[17] Kliger AS Foley RN Goldfarb DS, . KDOQI US commentary on the 2012 KDIGO clinical practice guideline for anemia in CKD. Am J Kidney Dis. 2013;62(5):849–859. doi:10.1053/j.ajkd.2013.06.008 23891356

[18] National Institute for Health and Care Excellence. Chronic Kidney Disease: Assessment and Management. 2023. Accessed May 13, 2024. https://www.nice.org.uk/guidance/ng203 34672500

[19] Rattanasompattikul M Feroze U Molnar MZ, . Charlson comorbidity score is a strong predictor of mortality in hemodialysis patients. Int Urol Nephrol. 2012;44(6):1813–1823. doi:10.1007/s11255-011-0085-9 22134841 PMC3595168

[20] Rattanasompattikul M Molnar MZ Zaritsky JJ, . Association of malnutrition–inflammation complex and responsiveness to erythropoiesis-stimulating agents in long-term hemodialysis patients. Nephrol Dial Transplant. 2013;28(7):1936–1945. doi:doi:10.1093/ndt/gfs368 23045431 PMC3707522

[21] Streja E Park J Chan T-Y, . Erythropoietin dose and mortality in hemodialysis patients: marginal structural model to examine causality. Int J Nephrol. 2016;2016:6087134. doi:10.1155/2016/6087134 27298736 PMC4889858

[22] Haase VH. Hypoxia-inducible factor–prolyl hydroxylase inhibitors in the treatment of anemia of chronic kidney disease. Kidney Int Suppl (2011). 2021;11(1):8–25. doi:10.1016/j.kisu.2020.12.002 33777492 PMC7983025

[23] Meadowcroft AM Cizman B Holdstock L, . Daprodustat for anemia: a 24-week, open-label, randomized controlled trial in participants on hemodialysis. Clin Kidney J. 2019;12(1):139–148. doi:10.1093/ckj/sfy014 30746141 PMC6366140

[24] Cizman B Sykes AP Paul G Zeig S Cobitz AR. An exploratory study of daprodustat in erythropoietin-hyporesponsive subjects. Kidney Int Rep. 2018;3(4):841–850. doi:10.1016/j.ekir.2018.02.009 29989040 PMC6035126

