# Supplementary material for: Association of Different Definitions of Erythropoiesis-Stimulating Agent Hyporesponsiveness with Major Adverse Cardiovascular Events: Insights from ASCEND-D
Source: Kidney360. 2025 May 7;6(9):1541–8. doi: 10.34067/KID.0000000808 (PMC12483048; doi:10.34067/KID.0000000808)
Supplement: Supplementary file 1 [file kidney360-6-01541-s001.pdf]

ASN American Society of Nephrology | JASN Journal of the American Society of Nephrology | KIDNEY360 Accessing Our World From Every Angle | CJASN Clinical Journal of the American Society of Nephrology

# ASN Journal Disclosure Form

As per ASN journal policy, I have disclosed any financial relationships or commitments I have held in the past 36 months as included below. I have listed my Current Employer below to indicate there is a relationship requiring disclosure. If no relationship exists, my Current Employer is not listed.

B. Claggett reports the following:
Employer: Harvard Medical School, Brigham and Women's Hospital; and Consultancy: Alnylam, Cardurion, Corvia, Cytokinetics, CVRx, Cardior, Eli Lilly, Intellia, Rocket.

I understand that the information above will be published within the journal article, if accepted, and that failure to comply and/or to accurately and completely report the potential financial conflicts of interest could lead to the following: 1) Prior to publication, article rejection, or 2) Post-publication, sanctions ranging from, but not limited to, issuing a correction, reporting the inaccurate information to the authors' institution, banning authors from submitting work to ASN journals for varying lengths of time, and/or retraction of the published work.

Name: Brian Claggett
Manuscript ID: K360-2024-000359R2
Manuscript Title: Association of Different Definitions of Erythropoiesis-Stimulating Agent Hyporesponsiveness with Major Adverse Cardiovascular Events: Insights From ASCEND-D
Date of Completion: November 11, 2024
Disclosure Updated Date: November 11, 2024

ASN American Society of Nephrology | JASN Journal of the American Society of Nephrology | KIDNEY360 Accessing Our World From Every Angle | CJASN Clinical Journal of the American Society of Nephrology

# ASN Journal Disclosure Form

As per ASN journal policy, I have disclosed any financial relationships or commitments I have held in the past 36 months as included below. I have listed my Current Employer below to indicate there is a relationship requiring disclosure. If no relationship exists, my Current Employer is not listed.

R. Correa-Rotter reports the following:
Employer: Instituto Nacional de Ciencias Médicas y Nutrición Salvador Zubiran, MEXICO and; Universidad Nacional Autónoma de México, MEXICO; Consultancy: Astra Zeneca, GSK, Boheringer Ingelheim, Bayer, Chinook, Novonordisk; Research Funding: Astra Zeneca, Novonordisk, Roche, Chinook, GSK; Honoraria: Amgen, Astra Zeneca, Boehringer Ingelheim, Sanofi, Bayer, Amgen; Advisory or Leadership Role: Membership Steering Committee of DAPA CKD, Astra Zeneca.; National Leader ASCEND study, GSK; National Leader FLOW study, Novonordisk, membership steering Committee FINE-REAL, Bayer.. Editorial Board Nefrologia Latinoamericana, Revista de Investigación Clinica, American Journal of Kidney Diseases. y Associate Editor: Blood Purification Associate Editor. Member of the Steering Committee of the World Kidney Day organization.; Speakers Bureau: Amgen, Astra Zeneca, Boehringer Ingelheim, Abbvie, Sanofi, Bayer, Novonordisk; and Other Interests or Relationships: Member of ASN, of International Society of Nephrology; Member of National Kidney Foundation; Member Mexican Institute for Research in Nephrology; Member Latin American Society of Nephrology and Hypertension; Member EDTA/ERA.

I understand that the information above will be published within the journal article, if accepted, and that failure to comply and/or to accurately and completely report the potential financial conflicts of interest could lead to the following: 1) Prior to publication, article rejection, or 2) Post-publication, sanctions ranging from, but not limited to, issuing a correction, reporting the inaccurate information to the authors' institution, banning authors from submitting work to ASN journals for varying lengths of time, and/or retraction of the published work.

Name: Ricardo Correa-Rotter
Manuscript ID: K360-2024-000359R2
Manuscript Title: Association of Different Definitions of Erythropoiesis-Stimulating Agent Hyporesponsiveness with Major Adverse Cardiovascular Events: Insights From ASCEND-D
Date of Completion: November 9, 2024
Disclosure Updated Date: November 9, 2024

ASN American Society of Nephrology | JASN Journal of the American Society of Nephrology | KIDNEY360 Accessing Our World From Every Angle | CJASN Clinical Journal of the American Society of Nephrology

# ASN Journal Disclosure Form

As per ASN journal policy, I have disclosed any financial relationships or commitments I have held in the past 36 months as included below. I have listed my Current Employer below to indicate there is a relationship requiring disclosure. If no relationship exists, my Current Employer is not listed.

L. Del Vecchio reports the following:
Employer: Sant?Anna Hospital, ASST Lariana, Como, Italy; Speakers Bureau: Speaker fees at meetings with indirect support of Astellas, Amgen, Vifor, Bayer, AstraZeneca, Boehringer; and Other Interests or Relationships: Member of the Council of the Italian Society of Nephrology; Member of the Steering Commettee of the EURECAm Working Group of ERA; Chairwoman of the Women of ERA Task Force.

I understand that the information above will be published within the journal article, if accepted, and that failure to comply and/or to accurately and completely report the potential financial conflicts of interest could lead to the following: 1) Prior to publication, article rejection, or 2) Post-publication, sanctions ranging from, but not limited to, issuing a correction, reporting the inaccurate information to the authors' institution, banning authors from submitting work to ASN journals for varying lengths of time, and/or retraction of the published work.

Name: Lucia Del Vecchio
Manuscript ID: K360-2024-000359R1
Manuscript Title: Association of Different Definitions of Erythropoiesis-Stimulating Agent Hyporesponsiveness with Major Adverse Cardiovascular Events: Insights From ASCEND-D
Date of Completion: September 7, 2024
Disclosure Updated Date: September 7, 2024

ASN American Society of Nephrology | JASN Journal of the American Society of Nephrology | KIDNEY360 Accessing Our World From Every Angle | CJASN Clinical Journal of the American Society of Nephrology

# ASN Journal Disclosure Form

As per ASN journal policy, I have disclosed any financial relationships or commitments I have held in the past 36 months as included below. I have listed my Current Employer below to indicate there is a relationship requiring disclosure. If no relationship exists, my Current Employer is not listed.

V. Kher reports the following:
Employer: Self employed; Consultancy: Torrent pharmaceuticals, India; Novartis , India; Roche, India; Panacea,India; Sanofi Aventis, India; Intas pharmaceuticals, India; Biocon pharmaceuticals , India ; GSK, India; RPG Life Sciences; Astra-zenaca , India, Zydus pharmaceuticals, India; Research Funding: Novartis India, Sanofi Aventis India,  Astellas India; Medtronics, zydus Pharmaceuticals, India; Honoraria: Novartis, India; Roche India ; Astellas, India; Torrent, India; Reddy's India; Intas India; JB pharmaceuticals India,; Advisory or Leadership Role: ROCHE INDIA, NOVARTIS INDIA, TORRENT, SANOFI AVENTIS,Reddys India, Biocon india Medtronics; Wockhardt, India, Zydus India, intas , India; Speakers Bureau: Novartis, India; Roche, India; Panacea, India; Sanofi Aventis, India; Intas, India; Biocon, India; Pfizer; Johnson and Johnson; JB pharmaceuticals; Astra-zenaca , India; Boehringer Ingelheim India, IPCA pharmaceuticals, India, Abbott , India.; and Other Interests or Relationships: Kidney health ,education and research society, India; Kidney education and research network  : non-profit NGOs; Regional coordinator research South Asia, International society of nephrology; Advisory board Indian Journal of Nephrology, Indian Journal of transplantation.

I understand that the information above will be published within the journal article, if accepted, and that failure to comply and/or to accurately and completely report the potential financial conflicts of interest could lead to the following: 1) Prior to publication, article rejection, or 2) Post-publication, sanctions ranging from, but not limited to, issuing a correction, reporting the inaccurate information to the authors' institution, banning authors from submitting work to ASN journals for varying lengths of time, and/or retraction of the published work.

Name: Vijay K. Kher
Manuscript ID: K360-2024-000359R2
Manuscript Title: Association of Different Definitions of Erythropoiesis-Stimulating Agent Hyporesponsiveness with Major Adverse Cardiovascular Events: Insights From ASCEND-D
Date of Completion: November 13, 2024
Disclosure Updated Date: November 13, 2024

ASN American Society of Nephrology
JASN Journal of the American Society of Nephrology
KIDNEY360 Accessing Our World From Every Angle
CJASN Clinical Journal of the American Society of Nephrology

# ASN Journal Disclosure Form

As per ASN journal policy, I have disclosed any financial relationships or commitments I have held in the past 36 months as included below. I have listed my Current Employer below to indicate there is a relationship requiring disclosure. If no relationship exists, my Current Employer is not listed.

F. McCausland reports the following:
Employer: Brigham and Women's Hospital; Consultancy: GlaxoSmithKline; Zydus Therapeutics Inc.; Research Funding: Research Funding paid to institution from NIDDK, Satellite Healthcare, Novartis, Lexicon, and Fifth Eye.; and Other Interests or Relationships: Expert witness fees from Rubin-Anders scientific.

I understand that the information above will be published within the journal article, if accepted, and that failure to comply and/or to accurately and completely report the potential financial conflicts of interest could lead to the following: 1) Prior to publication, article rejection, or 2) Post-publication, sanctions ranging from, but not limited to, issuing a correction, reporting the inaccurate information to the authors' institution, banning authors from submitting work to ASN journals for varying lengths of time, and/or retraction of the published work.

Name: Finnian R. McCausland
Manuscript ID: K360-2024-000359R2
Manuscript Title: Association of Different Definitions of Erythropoiesis-Stimulating Agent Hyporesponsiveness with Major Adverse Cardiovascular Events: Insights From ASCEND-D
Date of Completion: November 11, 2024
Disclosure Updated Date: March 6, 2024

ASN American Society of Nephrology | JASN Journal of the American Society of Nephrology | KIDNEY360 Accessing Our World From Every Angle | CJASN Clinical Journal of the American Society of Nephrology

# ASN Journal Disclosure Form

As per ASN journal policy, I have disclosed any financial relationships or commitments I have held in the past 36 months as included below. I have listed my Current Employer below to indicate there is a relationship requiring disclosure. If no relationship exists, my Current Employer is not listed.

G. McMahon reports the following:
Employer: Brigham and Women's Hospital; Consultancy: Optum Consulting; Advanced Instruments; Appellis Pharmaceuticals; Research Funding: Alexion Pharmaceuticals; Honoraria: Uptodate; and Other Interests or Relationships: Massachusetts Medical Society; Irish Nephrology Society.

I understand that the information above will be published within the journal article, if accepted, and that failure to comply and/or to accurately and completely report the potential financial conflicts of interest could lead to the following: 1) Prior to publication, article rejection, or 2) Post-publication, sanctions ranging from, but not limited to, issuing a correction, reporting the inaccurate information to the authors' institution, banning authors from submitting work to ASN journals for varying lengths of time, and/or retraction of the published work.

Name: Gearoid M. McMahon
Manuscript ID: K360-2024-000359R1
Manuscript Title: Association of Different Definitions of Erythropoiesis-Stimulating Agent Hyporesponsiveness with Major Adverse Cardiovascular Events: Insights From ASCEND-D
Date of Completion: September 3, 2024
Disclosure Updated Date: September 3, 2024

ASN
American Society of Nephrology
JASN
Journal of the American Society of Nephrology
KIDNEY360
Accessing Our World From Every Angle
CJASN
Clinical Journal of the American Society of Nephrology

# ASN Journal Disclosure Form

As per ASN journal policy, I have disclosed any financial relationships or commitments I have held in the past 36 months as included below. I have listed my Current Employer below to indicate there is a relationship requiring disclosure. If no relationship exists, my Current Employer is not listed.

K. Praditpornsilpa reports the following:
Employer: Chulalongkorn University; Research Funding: Fresenius Kabi; and Speakers Bureau: Fresenius Kabi.

I understand that the information above will be published within the journal article, if accepted, and that failure to comply and/or to accurately and completely report the potential financial conflicts of interest could lead to the following: 1) Prior to publication, article rejection, or 2) Post-publication, sanctions ranging from, but not limited to, issuing a correction, reporting the inaccurate information to the authors' institution, banning authors from submitting work to ASN journals for varying lengths of time, and/or retraction of the published work.

Name: Kearkiat Praditpornsilpa
Manuscript ID: K360-2024-000359R2
Manuscript Title: Association of Different Definitions of Erythropoiesis-Stimulating Agent Hyporesponsiveness with Major Adverse Cardiovascular Events: Insights From ASCEND-D
Date of Completion: November 11, 2024
Disclosure Updated Date: November 11, 2024

ASN American Society of Nephrology | JASN Journal of the American Society of Nephrology | KIDNEY360 Accessing Our World From Every Angle | CJASN Clinical Journal of the American Society of Nephrology

# ASN Journal Disclosure Form

As per ASN journal policy, I have disclosed any financial relationships or commitments I have held in the past 36 months as included below. I have listed my Current Employer below to indicate there is a relationship requiring disclosure. If no relationship exists, my Current Employer is not listed.

A. Rastogi reports the following:
Employer: UCLA; Consultancy: Akebia, Amicus, Ardelyx, Astrazeneca, Aurinia, Chiesi Global Inc., Chinook Therapeutics, Fresenius Medical Care-Vifor, GlaxoSmithKline, Novartis, Otsuka, Sanofi S. A., Travere Therapeutics, Vifor Pharma Inc.; Research Funding: Alnylam Pharmaceuticals, Astrazeneca , Bayer, GlaxoSmithKline, Idorsia Pharmaceuticals, Ltd, Kadmon Corporation, LLC, NIH, Novo Nordisk, Omeros Inc., Palladio Biosciences, Pfizer, Protalix Biotherapeutics Ltd, Reata Pharmaceuticals, Inc., Regulus Therapeutics, Summit Therapeutics, Sanofi; Honoraria: Amgen, Astrazeneca, Aurinia, Bayer, Baxter, Fresenius Medical Care, Genzyme/Sanofi, Janssen , Vifor Pharma Inc., Natera; Advisory or Leadership Role: Akebia, Amicus, Ardelyx, Astrazeneca, Aurinia, Chiesi Global Inc., Chinook Therapeutics, Fresenius Medical Care-Vifor, GlaxoSmithKline, Novartis, Otsuka, Sanofi S. A., Travere Therapeutics, Vifor Pharma Inc.; and Speakers Bureau: Amgen, Astrazeneca, Aurinia, Bayer, Baxter, Fresenius Medical Care, Genzyme/Sanofi, Vifor Pharma Inc., Natera.

I understand that the information above will be published within the journal article, if accepted, and that failure to comply and/or to accurately and completely report the potential financial conflicts of interest could lead to the following: 1) Prior to publication, article rejection, or 2) Post-publication, sanctions ranging from, but not limited to, issuing a correction, reporting the inaccurate information to the authors' institution, banning authors from submitting work to ASN journals for varying lengths of time, and/or retraction of the published work.

Name: Anjay Rastogi
Manuscript ID: K360-2024-000359R1
Manuscript Title: Association of Different Definitions of Erythropoiesis-Stimulating Agent Hyporesponsiveness with Major Adverse Cardiovascular Events: Insights From ASCEND-D
Date of Completion: September 3, 2024
Disclosure Updated Date: September 3, 2024

# ASN Journal Disclosure Form

As per ASN journal policy, I have disclosed any financial relationships or commitments I have held in the past 36 months as included below. I have listed my Current Employer below to indicate there is a relationship requiring disclosure. If no relationship exists, my Current Employer is not listed.

A. Singh reports the following:
Employer: Brigham and Women's Hospital; Harvard Medical School; Alexion/AZ; Consultancy: GSK, Bayer, Zydus, Nephrology Times, Chinook/Novartis, Alexion, Vera; Ownership Interest: AstraZeneca; Honoraria: GSK, Bayer, Zydus, Chinook/Novartis, Vera, Alexion; Advisory or Leadership Role: Nephrology Times; and Speakers Bureau: Bayer.

I understand that the information above will be published within the journal article, if accepted, and that failure to comply and/or to accurately and completely report the potential financial conflicts of interest could lead to the following: 1) Prior to publication, article rejection, or 2) Post-publication, sanctions ranging from, but not limited to, issuing a correction, reporting the inaccurate information to the authors' institution, banning authors from submitting work to ASN journals for varying lengths of time, and/or retraction of the published work.

Name: Ajay K. Singh
Manuscript ID: K360/2024/000359R2
Manuscript Title: Association of Different Definitions of Erythropoiesis Stimulating Agents Hyporesponsiveness with major Adverse Cardiovascular Events: Insights from Ascend D
Date of Completion: February 19, 2025
Disclosure Updated Date: February 19, 2025

ASN American Society of Nephrology | JASN Journal of the American Society of Nephrology | KIDNEY360 Accessing Our World From Every Angle | CJASN Clinical Journal of the American Society of Nephrology

# ASN Journal Disclosure Form

As per ASN journal policy, I have disclosed any financial relationships or commitments I have held in the past 36 months as included below. I have listed my Current Employer below to indicate there is a relationship requiring disclosure. If no relationship exists, my Current Employer is not listed.

S. Solomon reports the following:
Employer: Brigham and Women's Hospital; Consultancy: Abbott, Action, Akros, Alnylam, Amgen, Arena, AstraZeneca, Bayer, Boeringer-Ingelheim, BMS, Cardior, Cardurion, Corvia, Cytokinetics, Daiichi-Sankyo, GSK, Lilly, Merck, Myokardia, Novartis, Roche, Theracos, Quantum Genomics, Cardurion, Janssen, Cardiac Dimensions, Tenaya, Sanofi-Pasteur, Dinaqor, Tremeau, CellProThera, Moderna, American Regent, Sarepta, Lexicon, Anacardio, Akros, Valo; and Research Funding: Actelion, Alnylam, Amgen, AstraZeneca, Bellerophon, Bayer, BMS, Celladon, Cytokinetics, Eidos, Gilead, GSK, Ionis, Lilly, Mesoblast, MyoKardia, NIH/NHLBI, Neurotronik, Novartis, NovoNordisk, Respicardia, Sanofi Pasteur, Theracos, US2.AI, Edgewise.

I understand that the information above will be published within the journal article, if accepted, and that failure to comply and/or to accurately and completely report the potential financial conflicts of interest could lead to the following: 1) Prior to publication, article rejection, or 2) Post-publication, sanctions ranging from, but not limited to, issuing a correction, reporting the inaccurate information to the authors' institution, banning authors from submitting work to ASN journals for varying lengths of time, and/or retraction of the published work.

Name: Scott D. Solomon
Manuscript ID: K360-2024-000359R2
Manuscript Title: Association of Different Definitions of Erythropoiesis-Stimulating Agent Hyporesponsiveness with Major Adverse Cardiovascular Events: Insights From ASCEND-D
Date of Completion: January 23, 2025
Disclosure Updated Date: January 23, 2025

ASN American Society of Nephrology JASN Journal of the American Society of Nephrology KIDNEY360 Accessing Our World From Every Angle CJASN Clinical Journal of the American Society of Nephrology

# ASN Journal Disclosure Form

As per ASN journal policy, I have disclosed any financial relationships or commitments I have held in the past 36 months as included below. I have listed my Current Employer below to indicate there is a relationship requiring disclosure. If no relationship exists, my Current Employer is not listed.

O. Vieira Neto has nothing to disclose.

I understand that the information above will be published within the journal article, if accepted, and that failure to comply and/or to accurately and completely report the potential financial conflicts of interest could lead to the following: 1) Prior to publication, article rejection, or 2) Post-publication, sanctions ranging from, but not limited to, issuing a correction, reporting the inaccurate information to the authors' institution, banning authors from submitting work to ASN journals for varying lengths of time, and/or retraction of the published work.

Name: Osvaldo Merege Vieira Neto
Manuscript ID: K360-2024-000359R2
Manuscript Title: Association of Different Definitions of Erythropoiesis-Stimulating Agent Hyporesponsiveness with Major Adverse Cardiovascular Events: Insights From ASCEND-D
Date of Completion: November 12, 2024
Disclosure Updated Date: May 21, 2024

ASN American Society of Nephrology | JASN Journal of the American Society of Nephrology | KIDNEY360 Accessing Our World From Every Angle | CJASN Clinical Journal of the American Society of Nephrology

# ASN Journal Disclosure Form

As per ASN journal policy, I have disclosed any financial relationships or commitments I have held in the past 36 months as included below. I have listed my Current Employer below to indicate there is a relationship requiring disclosure. If no relationship exists, my Current Employer is not listed.

S. Waikar reports the following:
Employer: Boston University, Google (spouse); Consultancy: Wolters Kluewer, Bain, BioMarin, Goldfinch, GSK, Ikena, Strataca, Google, CANbridge, NovoNordisk, Ono, PepGen, Sironax, NovoNordisk, Vertex, Mineralys, Motric Bio; Research Funding: Vertex, Pfizer, JNJ, Natera; and Other Interests or Relationships: expert witness for litigation related to dialysis lab testing (Davita), PPIs (Pfizer), PFAO exposure (Dechert), voclosporin patent (Aurinia).

I understand that the information above will be published within the journal article, if accepted, and that failure to comply and/or to accurately and completely report the potential financial conflicts of interest could lead to the following: 1) Prior to publication, article rejection, or 2) Post-publication, sanctions ranging from, but not limited to, issuing a correction, reporting the inaccurate information to the authors' institution, banning authors from submitting work to ASN journals for varying lengths of time, and/or retraction of the published work.

Name: Sushrut S. Waikar
Manuscript ID: K360-2024-000359R1
Manuscript Title: Association of Different Definitions of Erythropoiesis-Stimulating Agent Hyporesponsiveness with Major Adverse Cardiovascular Events: Insights From ASCEND-D
Date of Completion: September 3, 2024
Disclosure Updated Date: May 24, 2024
