# Supplementary material for: Association of Different Definitions of Erythropoiesis-Stimulating Agent Hyporesponsiveness with Major Adverse Cardiovascular Events: Insights from ASCEND-D
Source: Kidney360. 2025 May 7;6(9):1541–8. doi: 10.34067/KID.0000000808 (PMC12483048; doi:10.34067/KID.0000000808)
Supplement: Supplementary file 2 [file kidney360-6-01541-s002.pdf]

Supplemental Material

Supplemental Table 1. Baseline characteristics according to ESA hyporesponsiveness status (Definition HypoR2)

Supplemental Table 2. Baseline characteristics according to ESA hyporesponsiveness status (Definition HypoR3)

Supplemental Table 3. Major Adverse Cardiovascular Events according to ESA hyporesponsiveness status (Unadjusted analyses)

Supplemental Table 4. Multivariable adjusted association of ESA hyporesponsiveness (Definition 1) with Major Adverse Cardiovascular Events

Supplemental Figure 1. Venn diagram illustrating overlap of patients between three definitions of ESA hyporesponsiveness.

Supplemental Figure 2. Restricted cubic spline showing the adjusted association of baseline log-transformed ERI with risk of MACE outcomes

Supplemental Table 1. Baseline characteristics according to ESA hyporesponsiveness status (Definition HypoR2)[a]

	Non-ESA hyporesponsiveness n=2353	ESA hyporesponsiveness n=573	
**Age, years**	58 ± 14	54 ± 15	p<0.001
**Female, n(%)**	977 (41.5%)	281 (49.0%)	p=0.001
**Race, n(%)**			p<0.001
**American Indian or Alaska Native**	26 (1.1 %)	17 (3.0 %)	
**Asian**	247 (10.5%)	104 (18.2%)	
**Black or African American**	338 (14.4%)	109 (19.0%)	
**Multiple**	45 (1.9 %)	17 (3.0 %)	
**Native Hawaiian or Other Pacific Islander**	40 (1.7 %)	11 (1.9 %)	
**White**	1657 (70.4%)	315 (55.0%)	
**Dialysis vintage, n(%)**			p=0.05
**0 to <2 years**	731 (31.1%)	154 (26.9%)	
**2 to <5 years**	848 (36.0%)	203 (35.4%)	
**≥5 years**	774 (32.9%)	216 (37.7%)	
**Hemodialysis, n(%)**	2118 (90.0%)	482 (84.1%)	p<0.001
**Access type, n(%)**			p<0.001
**AVF**	1700 (72.3%)	354 (61.8%)	
**AVG**	195 (8.3 %)	60 (10.5%)	
**Central Venous Catheter**	220 (9.4 %)	69 (12.0%)	
**Peritoneal Catheter**	235 (10.0%)	90 (15.7%)	
**Post-dialysis BMI at baseline, kg/m2**	27 [23 , 32]	25 [22 , 29]	p<0.001
**Systolic BP, mmHg**	133 ± 21	138 ± 23	p<0.001
**History of diabetes, n(%)**	998 (42.4%)	215 (37.5%)	p=0.03
**History of cardiovascular disease, n(%)**	1101 (46.8%)	226 (39.4%)	p=0.002
**Smoking status, n(%)**			p=0.45
**Current**	215 (9.1 %)	43 (7.5 %)	
**Former**	503 (21.4%)	122 (21.3%)	
**Never**	1635 (69.5%)	408 (71.2%)	

**Hemoglobin, g/dL**	10.4 ± 0.9	10.0 ± 1.0	p<0.001
**Albumin, g/dL**	3.9 ± 0.3	3.8 ± 0.4	p<0.001
**iPTH, ng/L**	311 [156 , 557]	353 [155 , 658]	p=0.004
**hsCRP, mg/L**	3.7 [1.5 , 9.8]	4.8 [1.9 , 13.4]	p<0.001
**Standardized prior ESA dose, U/week**	4813 [2941 , 6795]	16810 [12063 , 20303]	p<0.001
**Ferritin, ng/mL**	599 [344 , 964]	587 [344 , 941]	p=0.46
**Transferrin Saturation, %**	33 [26 , 42]	30 [24 , 38]	p<0.001
**Kt/V**	1.59 ± 0.37	1.63 ± 0.43	p=0.02
**Randomized to Daprodustat, n(%)**	1168 (49.6%)	299 (52.2%)	p=0.27

BMI, body mass index; BP, blood pressure; iPTH, intact parathyroid hormone; hsCRP, high sensitivity C-reactive protein; ESA, erythropoiesis-stimulating agent.

[a] Definition HypoR2 - an erythropoietin resistance index (ERI; calculated as the weight-adjusted weekly ESA dose, divided by the hemoglobin) ≥1.5U/kg/wk/g/L

Supplemental Table 2. Baseline characteristics according to ESA hyporesponsiveness status (Definition HypoR3)[a]

	Non-ESA hyporesponsiveness n=2345	ESA hyporesponsiveness n=581	
**Age, years**	58 ± 14	54 ± 14	p<0.001
**Female, n(%)**	999 (42.6%)	259 (44.6%)	p=0.39
**Race, n(%)**			p<0.001
**American Indian or Alaska Native**	22 (0.9 %)	21 (3.6 %)	
**Asian**	278 (11.9%)	73 (12.6%)	
**Black or African American**	312 (13.3%)	135 (23.2%)	
**Multiple**	46 (2.0 %)	16 (2.8 %)	
**Native Hawaiian or Other Pacific Islander**	32 (1.4 %)	19 (3.3 %)	
**White**	1655 (70.6%)	317 (54.6%)	
**Dialysis vintage, n(%)**			p=0.59
**0 to <2 years**	719 (30.7%)	166 (28.6%)	
**2 to <5 years**	840 (35.8%)	211 (36.3%)	
**≥5 years**	786 (33.5%)	204 (35.1%)	
**Hemodialysis, n(%)**	2104 (89.7%)	496 (85.4%)	p=0.003
**Access type, n(%)**			p<0.001
**AVF**	1688 (72.0%)	366 (63.1%)	
**AVG**	189 (8.1 %)	66 (11.4%)	
**Central Venous Catheter**	225 (9.6 %)	64 (11.0%)	
**Peritoneal Catheter**	241 (10.3%)	84 (14.5%)	
**Post-dialysis BMI at baseline, kg/m2**	27 [23 , 31]	27 [23 , 33]	p=0.02
**Systolic BP, mmHg**	133 ± 21	137 ± 23	p<0.001
**History of diabetes, n(%)**	957 (40.8%)	256 (44.1%)	p=0.16
**History of cardiovascular disease, n(%)**	1078 (46.0%)	249 (42.9%)	p=0.18
**Smoking status, n(%)**			p=0.01
**Current**	221 (9.4 %)	37 (6.4 %)	
**Former**	482 (20.6%)	143 (24.6%)	
**Never**	1642 (70.0%)	401 (69.0%)	

**Hemoglobin, g/dL**	10.4 ± 1.0	10.2 ± 1.0	p<0.001
**Albumin, g/dL**	3.9 ± 0.4	3.8 ± 0.4	p<0.001
**iPTH, ng/L**	313 [156 , 561]	342 [151 , 645]	p=0.03
**hsCRP, mg/L**	3.6 [1.4 , 9.4]	5.9 [2.5 , 14.5]	p<0.001
**Standardized prior ESA dose, U/week**	4788 [2940 , 6733]	16906 [12899 , 20536]	p<0.001
**Ferritin, ng/mL**	601 [347 , 966]	584 [335 , 941]	p=0.37
**Transferrin Saturation, %**	33 [26 , 42]	30 [24 , 38]	p<0.001
**Kt/V**	1.60 ± 0.37	1.57 ± 0.43	p=0.20
**Randomized to Daprodustat, n(%)**	1168 (49.8%)	299 (51.5%)	p=0.48

BMI, body mass index; BP, blood pressure; iPTH, intact parathyroid hormone; hsCRP, high sensitivity C-reactive protein; ESA, erythropoiesis-stimulating agent.

[a] Definition HypoR3 - baseline ESA dose (U/week) in top 20th percentile.

Supplemental Table 3. Major Adverse Cardiovascular Events according to ESA hyporesponsiveness status (Unadjusted analyses)

Outcome	ESA hyporesponsiveness		Non-ESA hyporesponsiveness		Hazard Ratio (95% CI)	P-value
	No. events/No. pts (%)	Rate /100 pt yrs (95%CI)	No. Events/No. pts (%)	Rate /100 pt yrs (95%CI)		
**Definition 1**						
**MACE composite**	117/363 (32%)	15.3 (12.8, 18.3)	635/2563 (25%)	10.8 (10.0, 11.7)	1.43 (1.17, 1.74)	<0.001
**All-cause death**	96/363 (26%)	11.9 (9.7, 14.5)	485/2563 (19%)	7.9 (7.2, 8.6)	1.54 (1.24, 1.91)	<0.001
**Nonfatal MI**	35/363 (10%)	4.5 (3.3, 6.3)	197/2563 (8%)	3.3 (2.9, 3.8)	1.36 (0.95, 1.95)	0.09
**Nonfatal stroke**	7/363 (2%)	0.9 (0.4, 1.8)	62/2563 (2%)	1.0 (0.8, 1.3)	0.85 (0.39, 1.85)	0.68
**Definition 2**						
**MACE composite**	174/573 (30%)	14.0 (12.0, 16.2)	578/2353 (25%)	10.7 (9.9, 11.6)	1.31 (1.11, 1.56)	0.002
**All-cause death**	142/573 (25%)	10.9 (9.2, 12.8)	439/2353 (19%)	7.8 (7.1, 8.5)	1.42 (1.17, 1.71)	<0.001
**Nonfatal MI**	52/573 (9%)	4.2 (3.2, 5.4)	180/2353 (8%)	3.3 (2.8, 3.8)	1.26 (0.92, 1.71)	0.15
**Nonfatal stroke**	9/573 (2%)	0.7 (0.4, 1.3)	60/2353 (3%)	1.1 (0.8, 1.4)	0.64 (0.32, 1.29)	0.21
**Definition 3**						
**MACE composite**	183/581 (31%)	14.6 (12.6, 16.8)	569/2345 (24%)	10.5 (9.7, 11.5)	1.39 (1.17, 1.64)	<0.001
**All-cause death**	145/581 (25%)	10.9 (9.2, 12.8)	436/2345 (19%)	7.7 (7.0, 8.5)	1.42 (1.17, 1.71)	<0.001
**Nonfatal MI**	56/581 (10%)	4.4 (3.4, 5.7)	176/2345 (8%)	3.2 (2.8, 3.7)	1.36 (1.01, 1.84)	0.04
**Nonfatal stroke**	12/581 (2%)	0.9 (0.5, 1.6)	57/2345 (2%)	1.0 (0.8, 1.3)	0.88 (0.47, 1.64)	0.68

Definition HypoR1 - an erythropoietin resistance index (ERI; calculated as the weight-adjusted weekly ESA dose, divided by the hemoglobin) of ≥2U/kg/wk/g/L or prior ESA dose/estimated dry weight ≥450 U/kg/wk; Definition HypoR2 - ERI ≥1.5U/kg/wk/g/L; Definition HypoR3 - baseline ESA dose (U/week) in top 20th percentile.

Supplemental Table 4. Multivariable adjusted association of ESA hyporesponsiveness (Definition 1) with Major Adverse Cardiovascular Events

	Full Model		Stepwise model	
**Variable**	**|Z| score**	**Hazard Ratio (95%CI)**	**|Z| score**	**Hazard Ratio (95%CI)**
**ESA Hyporesponsiveness (vs. not)**	2.28	1.32 (1.04, 1.68)	3.82	1.50 (1.22, 1.86)
**Randomized to Daprodustat (vs. ESA)**	0.75	0.94 (0.81, 1.10)		
**Age, per year**	8.90	1.03 (1.02, 1.04)	9.57	1.03 (1.02, 1.04)
**Female (vs. male)**	3.24	0.75 (0.63, 0.89)	3.75	0.74 (0.64, 0.87)
**Race**				
**American Indian or Alaska Native**	0.48	0.84 (0.41, 1.72)	0.70	0.80 (0.44, 1.48)
**Asian**	0.64	1.18 (0.72, 1.93)	1.59	1.41 (0.92, 2.17)
**Black or African American**	2.32	0.75 (0.59, 0.96)	2.26	0.77 (0.61, 0.97)
**Multiple**	2.42	2.20 (1.16, 4.16)	2.05	1.71 (1.03, 2.86)
**Native Hawaiian or Other Pacific Islander**	2.11	1.74 (1.04, 2.92)	2.42	1.77 (1.11, 2.81)
**White**	-	Ref	-	Ref
**History of Diabetes**	4.31	1.47 (1.23, 1.75)	4.17	1.39 (1.19, 1.62)
**History of Cardiovascular disease**	7.95	2.02 (1.70, 2.41)	8.32	1.98 (1.68, 2.32)
**Smoking status**				
**Current**	-	Ref	-	Ref
**Former**	2.56	0.68 (0.51, 0.92)	2.53	0.71 (0.54, 0.93)
**Never**	3.87	0.59 (0.45, 0.77)	4.13	0.59 (0.46, 0.76)
**Dialysis vintage**				
**0 to <2 years**	-	Ref		
**2 to <5 years**	0.65	0.94 (0.77, 1.14)		
**≥5 years**	0.93	1.10 (0.90, 1.34)		
**Dialysis access**				
**Arteriovenous fistula**	-	Ref	-	Ref
**Arteriovenous graft**	1.01	1.15 (0.88, 1.51)	0.46	1.06 (0.82, 1.38)
**Central Venous Catheter**	2.44	1.36 (1.06, 1.74)	3.11	1.42 (1.14, 1.78)
**Peritoneal Catheter**	0.11	1.02 (0.75,1.38)	1.01	0.87 (0.67, 1.14)
**Body mass index, per kg/m^2**	1.18	0.99 (0.98, 1.01)		
**Systolic BP, per 10 mmHg**	1.02	1.02 (0.98, 1.06)		

**Hemoglobin, per g/dL**	2.24	0.91 (0.83, 0.99)	2.29	0.91 (0.84, 0.99)
**Albumin, per 0.1 g/dL**	4.97	0.94 (0.92, 0.96)	6.75	0.93 (0.91, 0.95)
**Intact parathyroid hormone, per log ng/L**	1.16	1.04 (0.97, 1.12)		
**C-reactive protein, per log mg/L**	2.08	1.07 (1.00, 1.14)		
**Kt/V, per unit**	0.65	1.08 (0.86, 1.36)		
**Region**				
**Asia Pacific**	2.93	0.42 (0.23, 0.75)	3.27	0.43 (0.26, 0.71)
**Eastern Europe/South Africa**	3.19	0.68 (0.53, 0.86)	3.01	0.71 (0.57, 0.89)
**Western Europe/Canada/ANZ**	3.70	0.62 (0.48, 0.80)	3.76	0.65 (0.51, 0.81)
**Latin America**	0.01	1.00 (0.76, 1.33)	0.02	1.00 (0.77, 1.30)
**USA**	-	Ref	-	Ref

Definition HypoR1 - an erythropoietin resistance index (ERI; calculated as the weight-adjusted weekly ESA dose, divided by the hemoglobin) of ≥2U/kg/wk/g/L or prior ESA dose/estimated dry weight ≥450 U/kg/wk;

Supplemental Figure 1. Venn diagram illustrating overlap of patients between three definitions of ESA hyporesponsiveness.

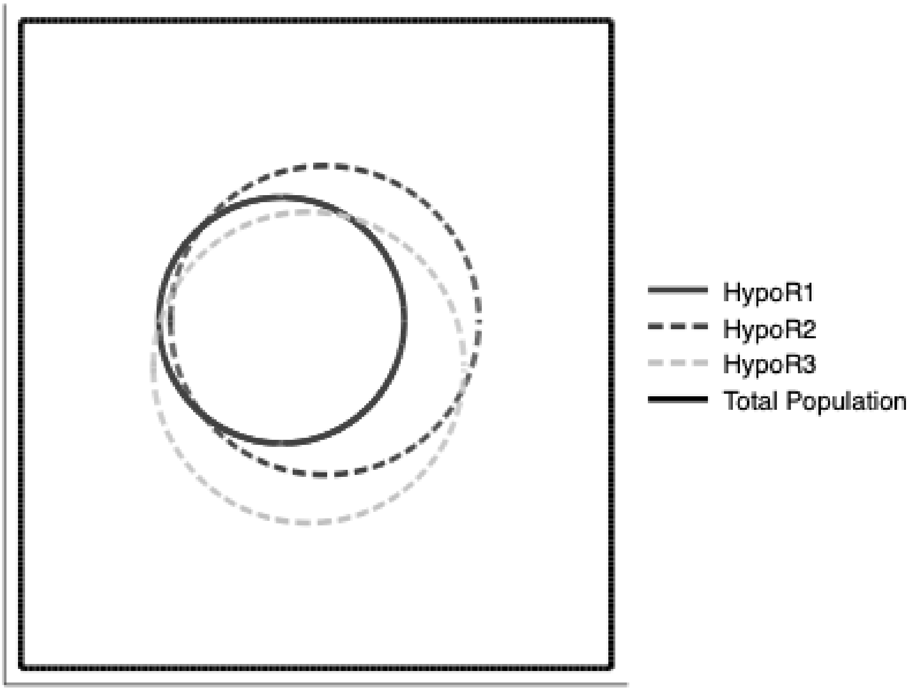

Supplemental Figure 2. Restricted cubic spline showing the adjusted association of baseline log-transformed ERI with risk of MACE outcomes

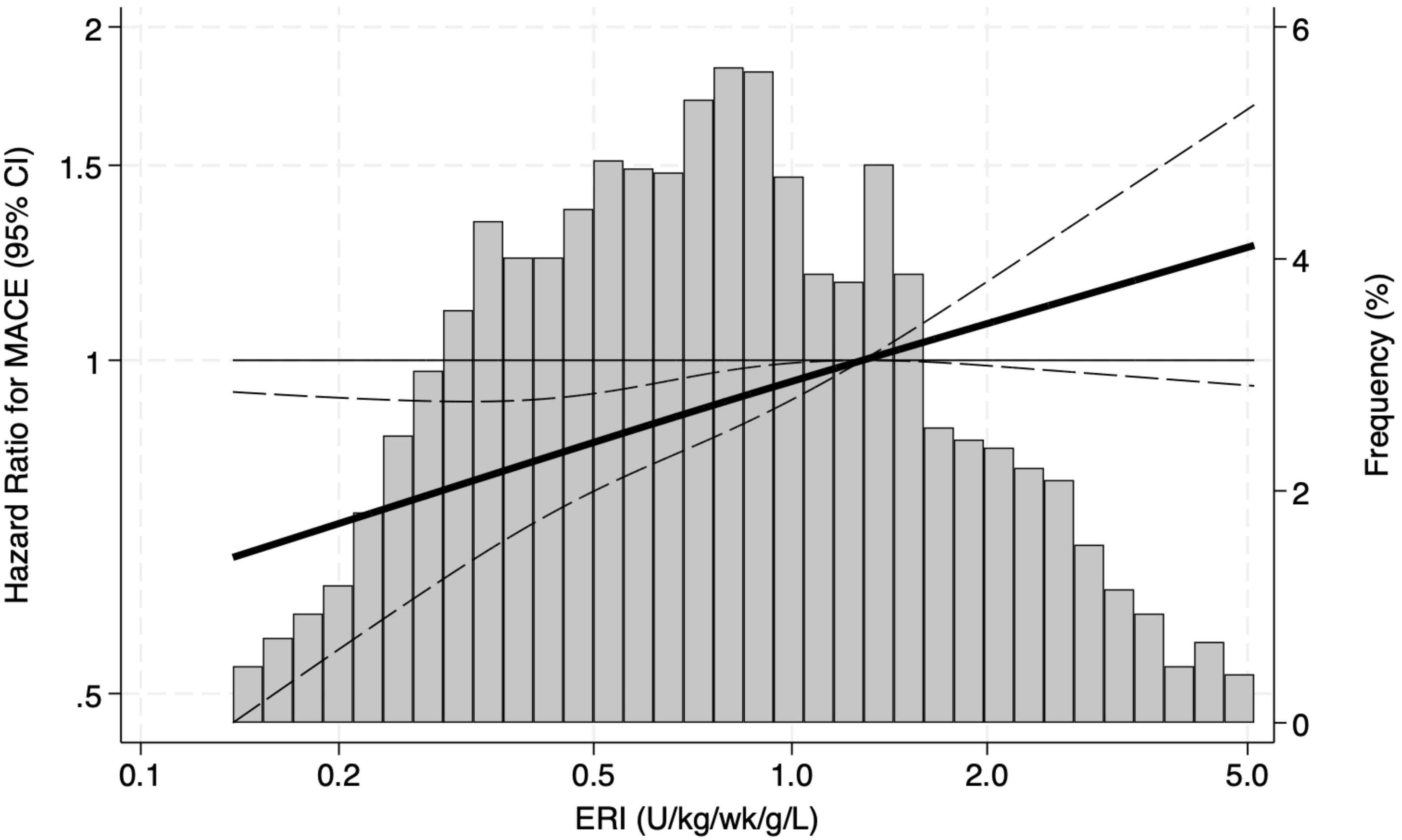

The solid line shows the hazard ratio; the dashed lines show the 95% confidence intervals; the histogram shows the frequency of individuals with a given baseline log-transformed erythropoietin resistance index.
